# Topological Water Network Analysis Around Amino Acids

**DOI:** 10.3390/molecules24142653

**Published:** 2019-07-22

**Authors:** Kwang-Eun Choi, Eunkyoung Chae, Anand Balupuri, Hye Ree Yoon, Nam Sook Kang

**Affiliations:** Graduate School of New Drug Discovery and Development, Chungnam National University, 99 Daehak-ro, Yuseong-gu, Daejeon 34134, Korea

**Keywords:** water, amino acids, molecular dynamics simulation, topological water network

## Abstract

Water molecules play a key role in protein stability, folding, function and ligand binding. Protein hydration has been studied using free energy perturbation algorithms. However, the study of protein hydration without free energy calculation is also an active field of research. Accordingly, topological water network (TWN) analysis has been carried out instead of free energy calculation in the present work to investigate hydration of proteins. Water networks around 20 amino acids in the aqueous solution were explored through molecular dynamics (MD) simulations. These simulation results were compared with experimental observations. Water molecules from the protein data bank structures showed TWN patterns similar to MD simulations. This work revealed that TWNs are effected by the surrounding environment. TWNs could provide valuable clues about the environment around amino acid residues in the proteins. The findings from this study could be exploited for TWN-based drug discovery and development.

## 1. Introduction

Proteins are composed of amino acids. Proteins in the biological environment are surrounded by water molecules which play crucial roles in the protein structure, function and dynamics [1,2]. Furthermore, they influence the binding process between biomacromolecular targets and small molecule ligands [3,4,5,6,7,8]. Water molecules within the active site of a protein can mediate protein-ligand interactions by bridging between protein and ligand or by being displaced upon complex formation. Water molecules have garnered the deserved attention in drug discovery research.

Over the past decade, considerable effort has been devoted to water-centric research. The studies involving water molecules are commonly focused on the free energy calculations. Several free energy-based algorithms are available, such as WaterMap (from Schrodinger) [9], SZMAP (from OpenEye) [10] and GIST (in Amber) [11]. However, free energy calculations are technically very demanding, time-consuming and require multiple simulations. They consider individual water molecules. These approaches are limited to discerning the energetics of hydration sites in the presence of a fixed distribution of surrounding water molecules. Hydrogen-bonded networks are often formed by water molecules in the binding site of the protein. The removal of one water molecule affects the free energies and structure of the remainder. The methods based on free energy calculations require extra simulations to account for these changes. Furthermore, the stability of the network as a whole cannot be understood well by knowing individual binding free energy of each water site. Besides, it is not possible to obtain information on cooperative effects between neighboring water molecules without additional analysis [12].

There is a paucity of water network-based research. Water networks, also known as water clusters, have been explored using infrared spectroscopy, Fourier-transform infrared spectroscopy, fluorescence spectroscopy, replica exchange molecular dynamics simulations and quantum calculations [13,14,15,16,17,18,19,20,21,22]. The experimental and theoretical studies involving water network analysis are mostly carried out on the pure water systems. Water networks arising from water molecules’ interactions with biomolecules have not been studied in detail thus far. This important research field is at an early stage of development. A recent study reported a novel procedure to quantify the disorder of extended water−water hydrogen-bond networks sampled in particle-based computer simulations. The strategy was depended on the conformational clustering of the hydrogen-bond connectivity states [23]. Another latest study showed the possibility of ligand discovery through water network analysis without free energy calculations [24]. They discovered new thermolysin inhibitors by water network analysis.

The authors have been involved in the research related to the water network analysis for several years. The authors developed an algorithm to determine the water-networks formed by hydrogen bonding without explicit free energy calculations [25,26,27,28]. Water networks form polygonal structures with 3-, 4-, 5- and 6-membered rings which the authors named as topological water networks (TWNs). As free energy calculations are technically very demanding, time-consuming and require multiple simulations [12], TWN analysis offers a better alternative to study the water networks. However, unlike free energy calculations, many studies have not been performed using the TWN analysis approach. Authors are exploring this approach in a number of different ways. Previously, the TWN results were correlated with the hydrophobic environment to explain kinase selectivity within systems including a variant of Gleevec and a series of substituted c-Jun N-terminal kinase (JNK) ligands [25]. Additionally, a method to compute the dipole moments of the water-ring network at specific positions in the adenosine triphosphate (ATP) binding pocket of kinase was proposed. The application of this method on two kinase systems (tyrosine-protein kinase ZAP-70/checkpoint kinase 1 and mitogen-activated protein kinase kinase kinase 5/3-phosphoinositide-dependent protein kinase-1) demonstrated that orientation of dipole moments played a crucial role in the protein-ligand binding mechanism [26]. TWN analysis was performed on several kinases to address the critical selectivity issue of kinase inhibitors. TWN analysis was further employed to develop a selective interleukin-1 receptor-associated kinase 4 (IRAK4) inhibitor [27]. Recently, the authors reported that TWNs influence the formation of α-strand/sheet structure in the α-synuclein [28]. This partially folded intermediate structure was proposed to be responsible for α-synuclein aggregation and fibril formation in the Parkinson’s disease. Furthermore, changes in the TWN pattern was observed under different conditions.

To date, several studies have reported the hydration pattern at the macromolecular (protein) level. However, the hydration pattern at the atomic level around the individual amino acids has not been studied yet. In this work, through molecular dynamics (MD) simulations, TWNs have been analyzed around all the amino acids at the atomic level for the first time. Furthermore, these results have been compared with TWNs of the X-ray crystallographic structures, which are available in the protein data bank (PDB). As amino acids are the components of proteins, TWN analysis around each amino acid provides novel insights into the amino acid specific hydration pattern at the atomic level.

## 2. Results and Discussion

### 2.1. Validation of MD Simulation Results for Each Amino Acid

**Figure 1:**
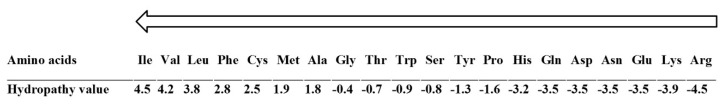
The hydropathy index of all amino acids according to the Kyte-Doolittle scale. The larger the value, the more hydrophobic is the amino acid. Ile is the most hydrophobic while Arg is the most hydrophilic. Amino acids are ordered from the most hydrophobic one (on the left hand side) to the most hydrophilic one (on the right hand side).

**Figure 2:**
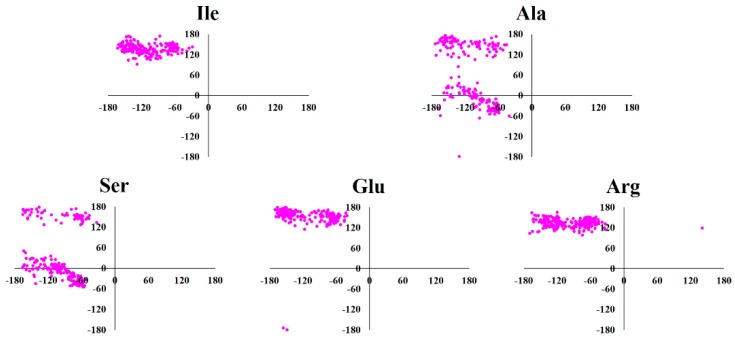
The Ramachandran plots for the representative amino acids. X-axis indicate φ torsion angle whereas Y-axis indicate ψ torsion angle. The φ and ψ torsion angles were extracted from the molecular dynamics (MD) trajectories. The representative amino acids were selected on the basis of hydropathy index (Ile—strong hydrophobic, Ala—weak hydrophobic, Ser—weak hydrophilic, Glu—strong hydrophilic and negative charge, and Arg—strong hydrophilic and positive charge).

Based on the hydrophobicity of amino acids proposed by Kyte-Doolittle (Figure 1) [29,30], MD simulation results of five representative amino acids (Ile—strong hydrophobic, Ala—weak hydrophobic, Ser—weak hydrophilic, Glu—strong hydrophilic and negative charge and Arg—strong hydrophilic and positive charge) were validated by the Ramachandran plot analysis. The Ramachandran plot has been the mainstay of protein structure validation for several years. It involves plotting of the backbone torsion angles (φ/ψ) for the amino acid residues. The φ and ψ values are plotted on the x-axis and y-axis, respectively, to predict the possible conformation of the amino acid. Torsion angles were computed using gmx rama module of GROMACS software [31]. This module selects the φ/ψ combinations from topology file and calculates these as a function of time. The Ramachandran plots for the representative amino acids are shown in Figure 2. It can be seen in the plots that torsion angles for the representative amino acids fell into well-defined regions of the Ramachandran plot. In accordance with previous studies [32,33], φ/ψ values were located in the right-handed α-helix region (lower left quadrant) and β-sheet region (upper left quadrant). It is evident from the Ramachandran plots that amino acid structures were correct. In addition, root-mean square deviation (RMSD) from the initial structure was calculated and plotted against simulation time to examine the dynamic stability of the amino acid structures. The RMSDs of all the amino acids (Supplementary Figure S1) remained stable with low values (<1 Å) throughout the MD simulation. The reasonably stabilized RMSD curves for the simulated amino acids suggest that they are suitable for further analysis.

### 2.2. TWN Pattern Around Amino Acids

Water molecules influence the structure and function of biomolecules. The concept of the hydration shell has been used to describe the properties of water around the biomolecules [34,35,36]. However, defining a hydration shell and differentiating it from the bulk water is challenging. It is usually defined as first water layer or sometimes a few water layers surrounding the biomolecule. The hydration shell consists of all those water molecules whose properties are significantly affected by the presence of biomolecule. The hydration shell thickness around a given site is usually provided as the distance from that site. The distance ranges of 2.5–4.0 and 5–7 Å are considered for the first and second hydration shells, respectively [37]. The hydration shell with one to two inner layers of water molecules was reported as a distance of 10 Å from the protein in an experimental study [20]. In the case of studies involving analysis of a single water molecule as one unit, it is possible to investigate the dynamics of water in multiple layers of the hydration shell. However, TWN has been explored as one unit instead of a single water molecule analysis in the present study. As explained before, TWN are formed due to hydrogen bonding among several water molecules. Water molecules participating in TWNs can be present in different layers of the shell. Consequently, in accordance with a previous study [20], only one hydration shell with a diameter of 10 Å from the amino acid coordinates was defined in this study. A distance of 10 Å was used for hydration shell as this value can include both first and second hydration shells. Water molecules present within 10 Å of the amino acid coordinates every 10 ps were extracted as well as every 5 ps for each simulated system. The TWN analysis was carried out on the extracted water molecules using the in-house Java and R codes.

**Figure 3:**
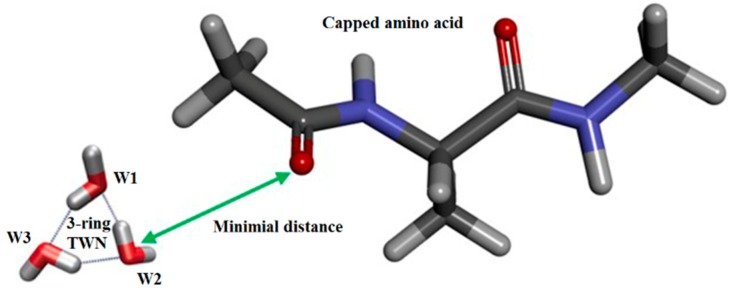
An illustration of a 3-ring topological water network (TWN) closely located near the O atom of an amino acid during the MD simulation. Water molecules involved in the 3-ring TWN are shown as W1-W3. TWNs derived from simulations were defined on the basis of non-covalent bond energy (−2.25 kcal mol^−1^).

Initially, the minimal distance between the TWN and amino acid atoms was calculated every 10 ps throughout the 1 ns MD simulation (Figure 3). As shown in Table 1 and Supplementary Table S1, the total number of TWNs decreased with the increase in the size of the TWN ring. The largest number of TWNs were observed for the 3-ring whereas the smallest number of TWNs were observed for 6-ring in the case of all the amino acids. This is due to the limited size of the hydration shell (distance of 10 Å) which can accommodate a low number of 6-ring TWNs as they are larger structures as compared to the 3-ring TWNs. As can be seen in Table 1, 3-ring TWNs were largely formed around the polar atoms (O or N) for hydrophilic amino acids, such as Asp, Asn and Glu, while they were mostly formed around the non-polar C atoms for hydrophobic amino acids, such as Ile and Phe. The total number of TWNs decreased with the increase in the size of the TWN ring. However, the frequency of 4- and 5-ring TWNs (Supplementary Table S1) showed similar patterns for the amino acids as the 3-ring TWNs. In the case of the residues with positively charged side chain (Lys and Arg), the TWN pattern was weaker compared to other strong hydrophilic resides, such as Gln, Asp, Asn and Glu. These results suggested that charges on the side chains of residues influence the TWN pattern. For the 6-ring (Supplementary Table S1), a somewhat similar TWN pattern was observed as obtained for the 3-, 4- and 5-rings. This might be due to the formation of very few 6-ring TWNs.

Additionally, the TWNs for backbone and side chain of each amino acid were separated. As can be seen in Table 1 and Supplementary Table S1, there is not much difference in the TWN frequency around the backbone atoms. In contrast, the side chain atoms of hydrophobic and hydrophilic residues showed considerable differences in the frequency of TWNs around them. Unlike hydrophilic amino acids, TWNs were largely formed around the side chain non-polar C atoms of hydrophobic amino acids. The side chain polar atoms (O or N) of hydrophilic amino acids showed high TWN frequency around them. However, due to the absence of polar atoms (O or N) in the side chains of hydrophobic amino acids, TWNs could not be observed around them. The separated backbone and side chain TWN results suggested that side chains were mainly responsible for the difference in the TWN pattern around the hydrophilic and hydrophobic amino acids.

In addition to 10 ps TWN analysis, the minimal distance between the TWN and amino acid atoms was calculated every 5 ps throughout the 1 ns MD simulation to check the consistency of the results. As shown in Supplementary Table S2, the 5 ps trajectories exhibited similar TWN patterns as the 10 ps trajectories. TWNs (3-, 4- and 5-ring) were mostly formed around the polar atoms (O or N) of hydrophilic amino acids, whereas they were mainly formed around the non-polar C atoms of hydrophobic amino acids. Similar to 10 ps TWN results, the weaker TWN pattern was observed for Lys and Arg which might be due to their positively charged side chains. The separated backbone and side chain TWN results showed similar patterns as observed for the 10 ps analysis. Similar TWN patterns for different time periods (10 ps and 5 ps) indicated the consistency of our results.

### 2.3. TWN Pattern for Residues of the PDB Structures

**Figure 4:**
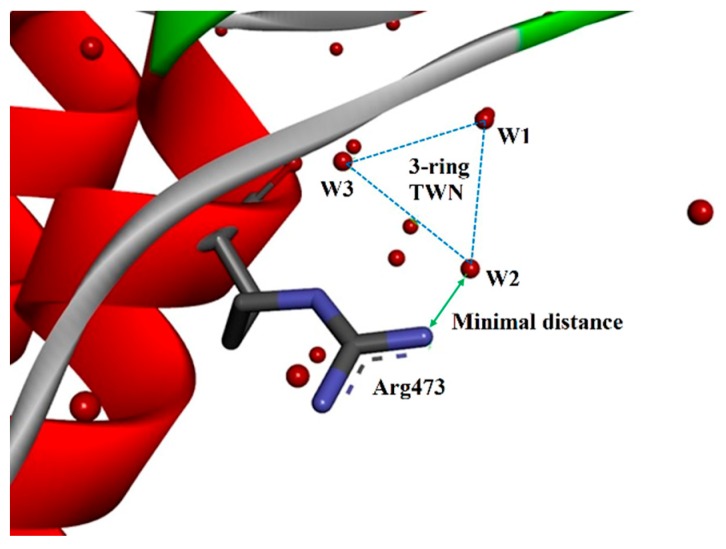
An illustration of a 3-ring TWN closely located near one of the N atoms of Arg473 in chain A of protein data bank (PDB) 2G1T (a Bcr-Abl tyrosine kinase structure). Water molecules are represented by red spheres. Water molecules involved in the 3-ring TWN are indicated as W1-W3. TWNs in PDBs were not extracted as energies but distances of <3.5 Å between the crystal water oxygen atoms due to the absence of hydrogen atoms in most of the PDBs.

In addition to the individual amino acid MD simulations, TWN patterns of the bonded amino acid residues of proteins were analyzed. As discussed in the methodology section, TWN analysis was carried out on the selected X-ray crystallographic structures available in the PDB. The hydrogen atoms are missing in most of the PDB files except for extremely high resolution crystal structures. Thus, TWNs for the crystal water molecules were calculated on the basis of maximum distance of <3.5 Å between the crystal water oxygen atoms (Figure 4) instead of the energies [38,39]. The TWN results (Table 2) for the filtered PDB dataset containing 16,548 structures demonstrated a similar pattern as observed in the MD simulations.

In the MD simulations (Table 1 and Supplementary Tables S1 and S2), total number of TWNs were almost similar around both the hydrophobic and hydrophilic residues because they were exposed to surrounding water molecules in the same way in the simulation box. However, individual amino acids are not the same as amino acid residues present in a protein. As shown in Table 2 and Supplementary Table S3, the total TWNs around the hydrophilic amino acid residues were found to be comparatively more than the hydrophobic amino acid residues. This could be due to hydrophilic residues being often more exposed to bulk water compared to the hydrophobic residues in the biological systems. Although total number of TWNs around hydrophobic residues was lower, the frequency of TWNs (3-, 4- and 5-rings) near their non-polar C atoms was comparatively higher than the non-polar C atoms of hydrophilic residues. Conversely, the frequency of TWNs was relatively higher near polar atoms (O or N) of the hydrophilic residues. As observed in the MD simulations, the total number of 6-ring TWNs in PDBs was also very small and the TWN pattern was slightly disordered accordingly. The TWN results of PDB structures were found to be consistent with MD simulation results. Similar to MD simulation results, the weaker TWN pattern was observed for positively charged residues particularly for Lys. Unlike other strong hydrophilic residues, the higher TWN frequency was observed near the non-polar C atoms of Lys. This might be due to its positively charged side chain. The TWN results of both MD simulations and PDB structures indicate that the charge effects the formation of these water networks.

As can be seen in Table 2 and Supplementary Table S3, the separated backbone and side chain TWN results for PDB structures were similar to the MD simulation results. The TWN frequency around the backbone atoms were almost similar, however, the side chain atoms showed significant differences. In accordance with the MD simulation results, TWNs were mainly formed around the side chain non-polar C atoms of the hydrophobic amino acids, whereas they were largely present around the side chain polar atoms (O or N) of the hydrophilic amino acids. The side chains were mainly responsible for the difference in the TWN pattern around the hydrophilic and hydrophobic amino acids.

Previously, another research group studied the electronic structure of the amino acids. They reported the atomic charge distribution for the amino acids [40]. The average charge on the C atoms of hydrophobic residues was found to be lower than the average charge on the C atoms of the hydrophilic residues. The charge differences could be responsible for the higher TWN frequency near the non-polar C atoms of the hydrophobic residues despite the low number of total TWNs around them. However, the lower average charge was observed around the C atoms of the Lys compared to the other hydrophilic residues, such as Asp, Asn and Glu. This could be the reason for the higher TWN frequency near non-polar C atoms of Lys.

## 3. Materials and Methods

### 3.1. Preparation and Capping of Amino Acids

**Figure 5:**
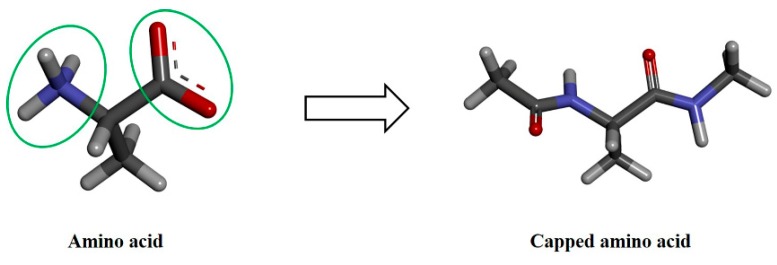
Amino acid capping at both termini. The amino acid is represented by the stick model where C, N, O and H atoms are shown in dark gray, blue, red and light gray colors, respectively. The capping regions are highlighted. Amino- and carboxyl-terminals were capped with acetyl (ACE) and N-methyl amide (NME) groups, respectively.

The 3D structures of 20 amino acids (Supplementary Figure S2) were built and the charge states were assigned by Discovery Studio 2017 R2 (BIOVIA, San Diego, CA, USA). In the case of His, both tautomeric structures namely Hsd (protonated at delta N) and Hse (protonated at epsilon N) were created. A previous study reported that capping of the termini of the amino acids ensured that the dynamics of the φ and ψ torsion angles were analogues to the dynamics within a peptide chain [32]. Accordingly, N and C termini of each amino acid were capped with acetyl (ACE) and N-methyl amide (NME) groups, respectively (Figure 5), for removing charge effects and imitating the peptide bond. The 3D structure of each amino acid was verified by a comparison with the energy minimized structure.

### 3.2. Molecular Dynamics (MD) Simulation

GROMACS software version 5.1 [31] with CHARMM27 all atom force field [41] was used to perform the MD simulations on all capped amino acid structures. The simulations on the charged amino acids were performed in a similar way as the neutral amino acids. Each amino acid was solvated in a cubic box of TIP3P water molecules [42] with a margin distance of 10 Å. The energy minimization of the system was carried out for 50,000 steps with the steepest descent method. The energy minimization was followed by NVT (constant number of particles, volume and temperature) and NPT (constant number of particles, pressure and temperature) equilibrations. The NVT equilibration was carried out for 100 ps at 298 K using Berendsen thermostat [43] to stabilize the temperature. The system was again equilibrated with NPT at a pressure of 1 bar for 100 ps using the Parrinello-Rahman barostat [44]. Finally, a production run was carried for 1 ns and coordinate trajectories were recorded every 5 ps. All the simulations were performed by applying periodic boundary conditions (PBC). All bond lengths were constrained using linear constraint solver (LINCS) algorithms [45]. The long-range interactions were handled using the particle mesh Ewald (PME) method [46], while short-range interactions were truncated at 10 Å.

### 3.3. Topological Water Network (TWN) Analysis

The authors developed an algorithm to determine the water-networks formed by hydrogen bonding without explicit free energy calculations. Water networks form polygonal structures which were named as TWNs. The computational protocol for TWN analysis is reported in their previous works [25,26,27,28]. TWNs refers to the hydrogen-bonded cyclic water-ring networks which are formed due to the hydrogen-bond interactions among the water molecules. TWNs include 3-, 4-, 5- and 6-membered rings. The potential functions considered in the TWNs involve a rigid TIP3P water model. Lennard-Jones and Coulomb potentials are commonly used to model interactions between water molecules [42]. The interaction potential energy between two water molecules is calculated using the following equation:

(1) $$v\left(a,b\right)=\overset{on\;a}{\underset{i}{\sum }}\overset{on\;b}{\underset{j}{\sum }}\frac{q_{i}q_{j}e^{2}}{r_{ij}}+\frac{A}{r_{oo}{}^{12}}-\frac{C}{r_{oo}{}^{6}}$$

where v(*a*, *b*) is the interaction potential energy between water molecules *a* and *b*, *r_oo_* indicates the distance between oxygen atoms, *q_i_* and *q_j_* represent partial charges on site *i* and *j*, respectively, *r_ij_* is the distance between charges *q_i_* and *q_j_*. A coulombic force between these two charges signifies the electrostatic attraction. A function that considers both attraction and repulsion simultaneously denotes the van der Waals interaction. The repulsive force of *i* and *j* is represented by parameter *A* whereas attraction is indicated by parameter *C*.

An energy criterion of −2.25 kcal mol^−1^ was carefully chosen to determine the hydrogen bond between water molecules as this value closely corresponds to the minimum of the pair-energy distribution of potential [42]. The parameters were selected in such a way that they produced reasonable structural and energetic results for liquid water. The parameters’ values are provided below:

*A* = 582,000 kcal Å^12^ mol^−1^ (2)

*C* = 595 kcal Å^6^ mol^−1^ (3)

*q_i_* = −0.834*e*, *q_j_* = 0.417*e* (4)

For TWN analysis, this study initially performed 1 ns MD simulation on each amino acid structure as discussed in the previous section. Afterwards, water molecules present within 10 Å of the alpha-carbon (C_α_) atoms of each residue were extracted every 10 ps as well as every 5 ps for each simulated system. Finally, the TWN analysis was carried out on the extracted water molecules using the equation provided above. Finally, the minimal distance between the TWN oxygen atom and heavy atoms of the residues was calculated. The extraction of water molecules, the TWN analysis and minimal distance calculations were performed using the in-house Java and R codes.

### 3.4. Analysis of PDB Structures

#### 3.4.1. Preparation of PDB Files

All the available PDB files (total of 126,292) were downloaded from the RCSB PDB website [47]. The non-protein PDB files, such as nucleic acids (DNA and RNA) and protein structures containing no crystal water molecules were removed. The structures (16,548 PDBs) having continuous hydration layer at the protein surface, i.e., X-ray structures refined against diffraction data with resolution better than 1.6 Å, were selected for the TWN analysis [48].

#### 3.4.2. TWN Analysis of Filtered PDB Files

As most of the 3D structures in the PDB contain no hydrogen atoms, the presence of an energetically significant hydrogen bond can be inferred when a probable donor and acceptor are within 3.5 Å of each other [38]. Accordingly, TWNs (3-, 4-, 5- and 6-membered rings) present in the PDB files were not extracted as energies but distances of <3.5 Å [39] between the crystal water oxygen atoms. Initially, all the water molecules present in the PDB structures were extracted and then distances between the crystal water oxygen atoms were analyzed to identify TWNs (distances of <3.5 Å). Then, a minimal distance between the TWN oxygen atom and heavy atoms of the residues was calculated. This study used in-house Java and R codes for the extraction of water molecules, the TWN analysis and minimal distance calculations.

## 4. Conclusions

The present work presents an algorithm to determine the water-networks formed by hydrogen bonding, without explicit free energy calculations. Water networks form polygonal structures with 3-, 4-, 5- and 6-membered rings which are known as TWNs. Herein, the frequency of the formation of TWNs near twenty amino acids were analyzed through MD simulations. Furthermore, the simulation results were compared with TWNs of PDBs. The results revealed that the formation of TWNs was affected by the environment around the amino acid residues in the protein. The TWN frequencies around the polar atoms (O or N) of hydrophilic residues were found to be higher compared to polar atoms (O or N) of the hydrophobic residues. On the contrary, the TWN frequencies were higher near the non-polar C atoms of the hydrophobic residues. Previously, several studies have reported hydration pattern of proteins at the macroscopic molecular level. However, to the authors’ knowledge, this is the first attempt to investigate a hydration pattern at the atomic level. Our simulations and calculations provide a novel insight into the amino acid specific hydration pattern at the atomic level for any protein. Furthermore, this work offers new perspectives to discover novel ligands as well as to optimize lead compounds in drug discovery considering a particular binding site environment.

## Figures and Tables

**Table 1:** The number of 3-ring TWNs observed around various atoms of all amino acids in the MD simulations. The TWN analysis was carried out on the water molecules which were extracted every 10 ps for each simulated system. The amino acids are ordered from the most hydrophobic one (Ile, on the left hand side) to the most hydrophilic one (Arg, on the right hand side), according to the Kyte-Doolitle scale.

(A) 3-ring TWNs (Backbone + Side chain)																					
	**Ile**	**Val**	**Leu**	**Phe**	**Cys**	**Met**	**Ala**	**Gly**	**Thr**	**Trp**	**Ser**	**Tyr**	**Pro**	**Hsd**	**Hse**	**Gln**	**Asp**	**Asn**	**Glu**	**Lys**	**Arg**
O	86	93	90	84	97	104	130	103	135	96	170	128	86	80	99	125	196	139	156	113	99
N	25	28	37	15	13	37	28	65	20	38	21	23	33	80	70	73	19	60	27	51	81
C	230	248	219	232	141	175	200	168	154	207	152	201	221	169	164	124	129	105	117	183	158
S					93	28															
Total	341	369	346	331	344	344	358	336	309	341	343	352	340	329	333	322	344	304	300	347	338
O,N/Total	0.33	0.33	0.37	0.30	0.32	0.41	0.44	0.50	0.50	0.39	0.56	0.43	0.35	0.49	0.51	0.61	0.63	0.65	0.61	0.47	0.53
C/Total	0.67	0.67	0.63	0.70	0.41	0.51	0.56	0.50	0.50	0.61	0.44	0.57	0.65	0.51	0.49	0.39	0.37	0.35	0.39	0.52	0.47
S/Total					0.27	0.08															
(B) 3-ring TWNs (Backbone)																					
	**Ile**	**Val**	**Leu**	**Phe**	**Cys**	**Met**	**Ala**	**Gly**	**Thr**	**Trp**	**Ser**	**Tyr**	**Pro**	**Hsd**	**Hse**	**Gln**	**Asp**	**Asn**	**Glu**	**Lys**	**Arg**
O	86	93	90	84	97	104	130	103	91	96	95	109	86	80	99	87	88	97	79	113	99
N	25	28	37	15	13	37	28	65	20	18	21	23	33	24	27	20	19	22	27	28	24
C	111	115	105	111	118	104	125	168	106	112	123	111	91	105	97	106	106	89	104	119	122
Total	222	236	232	210	228	245	283	336	217	226	239	243	210	209	223	213	213	208	210	260	245
O,N/Total	0.50	0.51	0.55	0.47	0.48	0.58	0.56	0.50	0.51	0.50	0.49	0.54	0.57	0.50	0.57	0.50	0.50	0.57	0.50	0.54	0.50
C/Total	0.50	0.49	0.45	0.53	0.52	0.42	0.44	0.50	0.49	0.50	0.51	0.46	0.43	0.50	0.43	0.50	0.50	0.43	0.50	0.46	0.50
(C) 3-ring TWNs (Side chain)																					
	**Ile**	**Val**	**Leu**	**Phe**	**Cys**	**Met**	**Ala**	**Gly**	**Thr**	**Trp**	**Ser**	**Tyr**	**Pro**	**Hsd**	**Hse**	**Gln**	**Asp**	**Asn**	**Glu**	**Lys**	**Arg**
O									44		75	19				38	108	42	77		
N										20				56	43	53		38		23	57
C	119	133	114	121	23	71	75		48	95	29	90	130	64	67	18	23	16	13	64	36
S					93	28															
Total	119	133	114	121	116	99	75		92	115	104	109	130	120	110	109	131	96	90	87	93
O,N/Total									0.48	0.17	0.72	0.17		0.47	0.39	0.83	0.82	0.83	0.86	0.26	0.61
C/Total	1.00	1.00	1.00	1.00	0.20	0.72	1.00		0.52	0.83	0.28	0.83	1.00	0.53	0.61	0.17	0.18	0.17	0.14	0.74	0.39
S/Total					0.80	0.28															

**Table 2:** The number of 3-ring TWNs observed around various atoms of all amino acids in the PDBs. The amino acids are ordered from the most hydrophobic one (Ile, on the left hand side) to the most hydrophilic one (Arg, on the right hand side), according to the Kyte-Doolitle scale.

(A) 3-ring TWNs (Backbone + Side chain)																				
	**Ile**	**Val**	**Leu**	**Phe**	**Cys**	**Met**	**Ala**	**Gly**	**Thr**	**Trp**	**Ser**	**Tyr**	**Pro**	**His**	**Gln**	**Asp**	**Asn**	**Glu**	**Lys**	**Arg**
O	3514	5045	6504	3003	918	1260	7254	8653	13110	1237	14400	8618	5832	1853	8862	29681	10018	28546	5174	4405
N	377	698	925	453	133	265	1259	1912	631	656	904	357	92	3733	3708	1305	4531	1038	8825	10522
C	591	813	980	578	33	338	1035	590	924	345	418	501	1627	785	450	351	329	776	1505	882
S					146	107														
Total	4482	6556	8409	4034	1230	1970	9548	11155	14665	2238	15722	9476	7551	6371	13020	31337	14878	30360	15504	15809
O,N/Total	0.87	0.88	0.88	0.86	0.85	0.77	0.89	0.95	0.94	0.85	0.97	0.95	0.78	0.88	0.97	0.99	0.98	0.97	0.90	0.94
C/Total	0.13	0.12	0.12	0.14	0.03	0.17	0.11	0.05	0.06	0.15	0.03	0.05	0.22	0.12	0.03	0.01	0.02	0.03	0.10	0.06
S/Total					0.12	0.05														
(B) 3-ring TWNs (Backbone)																				
	**Ile**	**Val**	**Leu**	**Phe**	**Cys**	**Met**	**Ala**	**Gly**	**Thr**	**Trp**	**Ser**	**Tyr**	**Pro**	**His**	**Gln**	**Asp**	**Asn**	**Glu**	**Lys**	**Arg**
O	3514	5045	6504	3003	918	1260	7254	8653	4959	1237	5402	2765	5832	1853	3062	5656	3866	5005	5174	4405
N	377	698	925	453	133	265	1259	1912	631	141	904	357	92	298	572	1305	882	1038	883	625
C	13	25	38	18	6	13	176	590	42	10	88	22	95	38	38	61	56	62	52	68
Total	3904	5768	7467	3474	1057	1538	8689	11155	5632	1388	6394	3144	6019	2189	3672	7022	4804	6105	6109	5098
O,N/Total	1.00	1.00	0.99	0.99	0.99	0.99	0.98	0.95	0.99	0.99	0.99	0.99	0.98	0.98	0.99	0.99	0.99	0.99	0.99	0.99
C/Total	0.00	0.00	0.01	0.01	0.01	0.01	0.02	0.05	0.01	0.01	0.01	0.01	0.02	0.02	0.01	0.01	0.01	0.01	0.01	0.01
(C) 3-ring TWNs (Side chain)																				
	**Ile**	**Val**	**Leu**	**Phe**	**Cys**	**Met**	**Ala**	**Gly**	**Thr**	**Trp**	**Ser**	**Tyr**	**Pro**	**His**	**Gln**	**Asp**	**Asn**	**Glu**	**Lys**	**Arg**
O									8151		8998	5853			5800	24025	6152	23541		
N										515				3435	3136		3649		7942	9897
C	578	788	942	560	27	325	859		882	335	330	479	1532	747	412	290	273	714	1453	814
S					146	107														
Total	578	788	942	560	173	432	859		9033	850	9328	6332	1532	4182	9348	24315	10074	24255	9395	10711
O,N/Total									0.90	0.61	0.96	0.92		0.82	0.96	0.99	0.97	0.97	0.85	0.92
C/Total	1.00	1.00	1.00	1.00	0.16	0.75	1.00		0.10	0.39	0.04	0.08	1.00	0.18	0.04	0.01	0.03	0.03	0.15	0.08
S/Total					0.84	0.25														

## Supplementary Materials

The following are available online, Table S1: The number of TWNs observed around various atoms of all amino acids in the MD simulations. TWN analysis was carried out on the water molecules which were extracted every 10 ps for each simulated system. Amino acids are ordered from the most hydrophobic one (Ile, on the left hand side) to the most hydrophilic one (Arg, on the right hand side), according to the Kyte-Doolitle scale. (A) 4-ring TWNs (Backbone + Side chain), (B) 4-ring TWNs (Backbone), (C) 4-ring TWNs (Side chain), (D) 5-ring TWNs (Backbone + Side chain), (E) 5-ring TWNs (Backbone), (F) 5-ring TWNs (Side chain), (G) 6-ring TWNs (Backbone + Side chain), (H) 6-ring TWNs (Backbone), (I) 6-ring TWNs (Side chain); Table S2: The number of TWNs observed around various atoms of all amino acids in the MD simulations. TWN analysis was carried out on the water molecules which were extracted every 5 ps for each simulated system. Amino acids are ordered from the most hydrophobic one (Ile, on the left hand side) to the most hydrophilic one (Arg, on the right hand side), according to the Kyte-Doolitle scale. (A) 3-ring TWNs (Backbone + Side chain), (B) 3-ring TWNs (Backbone), (C) 3-ring TWNs (Side chain), (D) 4-ring TWNs (Backbone + Side chain), (E) 4-ring TWNs (Backbone), (F) 4-ring TWNs (Side chain), (G) 5-ring TWNs (Backbone + Side chain), (H) 5-ring TWNs (Backbone), (I) 5-ring TWNs (Side chain), (J) 6-ring TWNs (Backbone + Side chain), (K) 6-ring TWNs (Backbone), (L) 6-ring TWNs (Side chain); Table S3: The number of TWNs observed around various atoms of all amino acids in the PDBs. Amino acids are ordered from the most hydrophobic one (Ile, on the left hand side) to the most hydrophilic one (Arg, on the right hand side), according to the Kyte-Doolitle scale. (A) 4-ring TWNs (Backbone + Side chain), (B) 4-ring TWNs (Backbone), (C) 4-ring TWNs (Side chain), (D) 5-ring TWNs (Backbone + Side chain), (E) 5-ring TWNs (Backbone), (F) 5-ring TWNs (Side chain), (G) 6-ring TWNs (Backbone + Side chain), (H) 6-ring TWNs (Backbone), (I) 6-ring TWNs (Side chain); Figure S1: RMSD plot for the backbone atoms of amino acids from the initial structures throughout the 1 ns MD simulation as a function of time; Figure S2: Structures of the amino acids studied in the present work.
