# Supplementary material for: Topological Water Network Analysis Around Amino Acids
Source: Molecules. 2019 Jul 22;24(14):2653. doi: 10.3390/molecules24142653 (PMC6681432; doi:10.3390/molecules24142653)
Supplement: Supplementary file 1 [file molecules-24-02653-s001.pdf]

Supplementary Materials

Topological Water Network Analysis Around Amino Acids

Kwang-Eun Choi ‡, Eunkyoung Chae ‡, Anand Balupuri, Hye Ree Yoon and Nam Sook Kang *

Graduate School of New Drug Discovery and Development, Chungnam National University, 99 Daehak-ro, Yuseong-gu, Daejeon 34134, Korea

Table S1. The number of TWNs observed around various atoms of all amino acids in the MD simulations. TWN analysis was carried out on the water molecules which were extracted every 10 ps for each simulated system. Amino acids are ordered from the most hydrophobic one (Ile, on the left hand side) to the most hydrophilic one (Arg, on the right hand side), according to the Kyte-Doolitle scale.

(A) 4-ring TWNs (Backbone + Side chain)

	**Ile**	**Val**	**Leu**	**Phe**	**Cys**	**Met**	**Ala**	**Gly**	**Thr**	**Trp**	**Ser**	**Tyr**	**Pro**	**Hsd**	**Hse**	**Gln**	**Asp**	**Asn**	**Glu**	**Lys**	**Arg**
O	61	52	68	70	69	73	75	66	101	66	112	73	55	67	56	78	105	105	105	71	79
N	25	12	20	13	23	26	15	50	19	31	12	20	22	49	55	46	10	51	27	37	43
C	157	132	139	143	80	114	132	118	100	122	95	124	143	120	119	79	74	78	55	120	100
S					47	28															
Total	243	196	227	226	219	241	222	234	220	219	219	217	220	236	230	203	189	234	187	228	222
O,N/Total	0.35	0.33	0.39	0.37	0.42	0.41	0.41	0.50	0.55	0.44	0.57	0.43	0.35	0.49	0.48	0.61	0.61	0.67	0.71	0.47	0.55
C/Total	0.65	0.67	0.61	0.63	0.37	0.47	0.59	0.50	0.45	0.56	0.43	0.57	0.65	0.51	0.52	0.39	0.39	0.33	0.29	0.53	0.45
S/Total					0.21	0.12															

(B) 4-ring TWNs (Backbone)

	**Ile**	**Val**	**Leu**	**Phe**	**Cys**	**Met**	**Ala**	**Gly**	**Thr**	**Trp**	**Ser**	**Tyr**	**Pro**	**Hsd**	**Hse**	**Gln**	**Asp**	**Asn**	**Glu**	**Lys**	**Arg**
O	61	52	68	70	69	73	75	103	67	66	69	67	55	67	56	65	48	63	60	71	79
N	25	12	20	13	23	26	15	65	19	14	12	20	22	21	27	18	10	16	27	26	15
C	73	59	68	67	63	81	78	168	59	69	77	74	68	73	79	71	63	64	48	72	66
Total	159	123	156	150	155	180	168	336	145	149	158	161	145	161	162	154	121	143	135	169	160
O,N/Total	0.54	0.52	0.56	0.55	0.59	0.55	0.54	0.50	0.59	0.54	0.51	0.54	0.53	0.55	0.51	0.54	0.48	0.55	0.64	0.57	0.59
C/Total	0.46	0.48	0.44	0.45	0.41	0.45	0.46	0.50	0.41	0.46	0.49	0.46	0.47	0.45	0.49	0.46	0.52	0.45	0.36	0.43	0.41

(C) 4-ring TWNs (Side chain)

	**Ile**	**Val**	**Leu**	**Phe**	**Cys**	**Met**	**Ala**	**Gly**	**Thr**	**Trp**	**Ser**	**Tyr**	**Pro**	**Hsd**	**Hse**	**Gln**	**Asp**	**Asn**	**Glu**	**Lys**	**Arg**
O									34		43	6				13	57	42	45		
N										17				28	28	28		35		11	28
C	84	73	71	76	17	33	54		41	53	18	50	75	47	40	8	11	14	7	48	34
S					47	28															
Total	84	73	71	76	64	61	54		75	70	61	56	75	75	68	49	68	91	52	59	62
O,N/Total									0.45	0.24	0.70	0.11		0.37	0.41	0.84	0.84	0.85	0.87	0.19	0.45
C/Total	1.00	1.00	1.00	1.00	0.27	0.54	1.00		0.55	0.76	0.30	0.89	1.00	0.63	0.59	0.16	0.16	0.15	0.13	0.81	0.55

	Ile	Val	Leu	Phe	Cys	Met	Ala	Gly	Thr	Trp	Ser	Tyr	Pro	Hsd	Hse	Gln	Asp	Asn	Glu	Lys	Arg
S/Total					0.73	0.46															

(D) 5-ring TWNs (Backbone + Side chain)

	**Ile**	**Val**	**Leu**	**Phe**	**Cys**	**Met**	**Ala**	**Gly**	**Thr**	**Trp**	**Ser**	**Tyr**	**Pro**	**Hsd**	**Hse**	**Gln**	**Asp**	**Asn**	**Glu**	**Lys**	**Arg**
O	27	29	17	26	25	31	18	33	37	23	54	20	30	27	31	35	44	36	34	28	36
N	4	5	10	9	10	11	8	17	8	11	0	9	5	16	25	12	7	24	6	20	19
C	52	45	49	51	39	40	48	35	30	39	15	35	55	36	38	21	19	25	30	36	25
S					23	14															
Total	83	79	76	86	97	96	74	85	75	73	69	64	90	79	94	68	70	85	70	84	80
O,N/Total	0.37	0.43	0.36	0.41	0.36	0.44	0.35	0.59	0.60	0.47	0.78	0.45	0.39	0.54	0.60	0.69	0.73	0.71	0.57	0.57	0.69
C/Total	0.63	0.57	0.64	0.59	0.40	0.42	0.65	0.41	0.40	0.53	0.22	0.55	0.61	0.46	0.40	0.31	0.27	0.29	0.43	0.43	0.31
S/Total					0.24	0.14															

(E) 5-ring TWNs (Backbone)

	**Ile**	**Val**	**Leu**	**Phe**	**Cys**	**Met**	**Ala**	**Gly**	**Thr**	**Trp**	**Ser**	**Tyr**	**Pro**	**Hsd**	**Hse**	**Gln**	**Asp**	**Asn**	**Glu**	**Lys**	**Arg**
O	27	29	17	26	25	31	18	33	21	23	35	19	30	27	31	28	20	23	17	28	36
N	4	5	10	9	10	11	8	17	8	8	0	9	5	7	12	5	7	8	6	12	11
C	19	19	28	20	34	21	29	35	18	16	12	15	26	19	27	16	12	19	26	23	19
Total	50	53	55	55	69	63	55	85	47	47	47	43	61	53	70	49	39	50	49	63	66
O,N/Total	0.62	0.64	0.49	0.64	0.51	0.67	0.47	0.59	0.62	0.66	0.74	0.65	0.57	0.64	0.61	0.67	0.69	0.62	0.47	0.63	0.71
C/Total	0.38	0.36	0.51	0.36	0.49	0.33	0.53	0.41	0.38	0.34	0.26	0.35	0.43	0.36	0.39	0.33	0.31	0.38	0.53	0.37	0.29

(F) 5-ring TWNs (Side chain)

	**Ile**	**Val**	**Leu**	**Phe**	**Cys**	**Met**	**Ala**	**Gly**	**Thr**	**Trp**	**Ser**	**Tyr**	**Pro**	**Hsd**	**Hse**	**Gln**	**Asp**	**Asn**	**Glu**	**Lys**	**Arg**
O									16		19	1				7	24	13	17		
N										3				9	13	7		16		8	8
C	33	26	21	31	5	19	19		12	23	3	20	29	17	11	5	7	6	4	13	6
S					23	14															
Total	33	26	21	31	28	33	19		28	26	22	21	29	26	24	19	31	35	21	21	14
O,N/Total									0.57	0.12	0.86	0.05		0.35	0.54	0.74	0.77	0.83	0.81	0.38	0.57
C/Total	1.00	1.00	1.00	1.00	0.18	0.58	1.00		0.43	0.88	0.14	0.95	1.00	0.65	0.46	0.26	0.23	0.17	0.19	0.62	0.43
S/Total					0.82	0.42															

(G) 6-ring TWNs (Backbone + Side chain)

	**Ile**	**Val**	**Leu**	**Phe**	**Cys**	**Met**	**Ala**	**Gly**	**Thr**	**Trp**	**Ser**	**Tyr**	**Pro**	**Hsd**	**Hse**	**Gln**	**Asp**	**Asn**	**Glu**	**Lys**	**Arg**
O	5	5	9	7	9	15	10	2	8	3	11	10	4	5	5	11	15	5	5	10	7
N	0	2	3	2	3	2	4	7	4	5	0	1	1	3	5	2	0	8	4	1	3
C	22	12	10	8	5	11	15	5	10	10	6	9	14	5	10	7	6	4	9	8	7
S					9	2															
Total	27	19	22	17	26	30	29	14	22	18	17	20	19	13	20	20	21	17	18	19	17
O,N/Total	0.19	0.37	0.55	0.53	0.46	0.57	0.48	0.64	0.55	0.44	0.65	0.55	0.26	0.62	0.50	0.65	0.71	0.76	0.50	0.58	0.59
C/Total	0.81	0.63	0.45	0.47	0.19	0.37	0.52	0.36	0.45	0.56	0.35	0.45	0.74	0.38	0.50	0.35	0.29	0.24	0.50	0.42	0.41
S/Total					0.35	0.06															

(H) 6-ring TWNs (Backbone)

	**Ile**	**Val**	**Leu**	**Phe**	**Cys**	**Met**	**Ala**	**Gly**	**Thr**	**Trp**	**Ser**	**Tyr**	**Pro**	**Hsd**	**Hse**	**Gln**	**Asp**	**Asn**	**Glu**	**Lys**	**Arg**
O	5	5	9	7	9	15	10	2	7	3	9	9	4	5	5	10	2	4	3	10	7
N	0	2	3	2	3	2	4	7	4	2	0	1	1	1	2	1	0	5	4	1	2
C	11	9	5	2	5	8	6	5	7	5	4	4	3	3	8	4	5	2	7	3	5
Total	16	16	17	11	17	25	20	14	18	10	13	14	8	9	15	15	7	11	14	14	14
O,N/Total	0.31	0.44	0.71	0.82	0.71	0.68	0.70	0.64	0.61	0.50	0.69	0.71	0.63	0.67	0.47	0.73	0.29	0.82	0.50	0.79	0.64
C/Total	0.69	0.56	0.29	0.18	0.29	0.32	0.30	0.36	0.39	0.50	0.31	0.29	0.38	0.33	0.53	0.27	0.71	0.18	0.50	0.21	0.36

(I) 6-ring TWNs (Side chain)

	**Ile**	**Val**	**Leu**	**Phe**	**Cys**	**Met**	**Ala**	**Gly**	**Thr**	**Trp**	**Ser**	**Tyr**	**Pro**	**Hsd**	**Hse**	**Gln**	**Asp**	**Asn**	**Glu**	**Lys**	**Arg**
O									1		2	1				1	13	1	2		
N										3				2	3	1		3			1
C	11	3	5	6		3	9		3	5	2	5	11	2	2	3	1	2	2	5	2
S					9	2															
Total	11	3	5	6	9	5	9		4	8	4	6	11	4	5	5	14	6	4	5	3
O,N/Total									0.25	0.38	0.50	0.17		0.50	0.60	0.40	0.93	0.67	0.50	0.00	0.33
C/Total	1.00	1.00	1.00	1.00	0.00	0.60	1.00		0.75	0.63	0.50	0.83	1.00	0.50	0.40	0.60	0.07	0.33	0.50	1.00	0.67
S/Total					1.00	0.40															

Table S2. The number of TWNs observed around various atoms of all amino acids in the MD simulations. TWN analysis was carried out on the water molecules which were extracted every 5 ps for each simulated system. Amino acids are ordered from the most hydrophobic one (Ile, on the left hand side) to the most hydrophilic one (Arg, on the right hand side), according to the Kyte-Doolitle scale.

(A) 3-ring TWNs (Backbone + Side chain)

	**Ile**	**Val**	**Leu**	**Phe**	**Cys**	**Met**	**Ala**	**Gly**	**Thr**	**Trp**	**Ser**	**Tyr**	**Pro**	**Hsd**	**Hse**	**Gln**	**Asp**	**Asn**	**Glu**	**Lys**	**Arg**
O	173	190	174	175	205	194	262	227	288	198	351	251	164	182	204	274	406	299	336	201	202
N	47	59	66	37	38	56	61	128	35	82	43	41	72	176	144	146	45	121	54	107	155
C	464	451	452	438	285	314	434	332	329	410	306	376	421	344	342	241	220	232	234	348	309
S					168	73															
Total	684	700	692	650	696	637	757	687	652	690	700	668	657	702	690	661	671	652	624	656	666
O,N/Total	0.32	0.36	0.35	0.33	0.35	0.40	0.43	0.52	0.50	0.41	0.56	0.44	0.36	0.51	0.50	0.64	0.67	0.64	0.62	0.47	0.54
C/Total	0.68	0.64	0.65	0.67	0.41	0.49	0.57	0.48	0.50	0.59	0.44	0.56	0.64	0.49	0.50	0.36	0.33	0.36	0.38	0.53	0.46
S/Total					0.24	0.11															

(B) 3-ring TWNs (Backbone)

	**Ile**	**Val**	**Leu**	**Phe**	**Cys**	**Met**	**Ala**	**Gly**	**Thr**	**Trp**	**Ser**	**Tyr**	**Pro**	**Hsd**	**Hse**	**Gln**	**Asp**	**Asn**	**Glu**	**Lys**	**Arg**
O	173	190	174	175	205	194	262	227	198	198	206	213	164	182	204	185	189	198	177	201	202
N	47	59	66	37	38	56	61	128	35	35	43	41	72	53	49	53	45	35	54	54	52
C	223	195	218	214	225	195	275	332	213	227	241	205	182	202	194	197	179	193	202	217	228
Total	443	444	458	426	468	445	598	687	446	460	490	459	418	437	447	435	413	426	433	472	482
O,N/Total	0.50	0.56	0.52	0.50	0.52	0.56	0.54	0.52	0.52	0.51	0.51	0.55	0.56	0.54	0.57	0.55	0.57	0.55	0.53	0.54	0.53
C/Total	0.50	0.44	0.48	0.50	0.48	0.44	0.46	0.48	0.48	0.49	0.49	0.45	0.44	0.46	0.43	0.45	0.43	0.45	0.47	0.46	0.47

(C) 3-ring TWNs (Side chain)

	**Ile**	**Val**	**Leu**	**Phe**	**Cys**	**Met**	**Ala**	**Gly**	**Thr**	**Trp**	**Ser**	**Tyr**	**Pro**	**Hsd**	**Hse**	**Gln**	**Asp**	**Asn**	**Glu**	**Lys**	**Arg**
O									90		145	38				89	217	101	159		
N										47				123	95	93		86		53	103
C	241	256	234	224	60	119	159		116	183	65	171	239	142	148	44	41	39	32	131	81
S					168	73															
Total	241	256	234	224	228	192	159		206	230	210	209	239	265	243	226	258	226	191	184	184
O,N/Total									0.44	0.20	0.69	0.18		0.46	0.39	0.81	0.84	0.83	0.83	0.29	0.56
C/Total	1.00	1.00	1.00	1.00	0.26	0.62	1.00		0.56	0.80	0.31	0.82	1.00	0.54	0.61	0.19	0.16	0.17	0.17	0.71	0.44
S/Total					0.74	0.38															

(D) 4-ring TWNs (Backbone + Side chain)

	**Ile**	**Val**	**Leu**	**Phe**	**Cys**	**Met**	**Ala**	**Gly**	**Thr**	**Trp**	**Ser**	**Tyr**	**Pro**	**Hsd**	**Hse**	**Gln**	**Asp**	**Asn**	**Glu**	**Lys**	**Arg**
O	117	106	138	131	132	146	158	148	199	141	228	153	110	133	124	155	232	201	232	143	143
N	44	25	36	41	44	45	41	109	34	69	31	42	49	93	101	112	25	93	42	63	94
C	312	273	270	291	170	220	257	220	179	233	174	250	302	233	225	169	151	165	121	238	189
S					111	51															
Total	473	404	444	463	457	462	456	477	412	443	433	445	461	459	450	436	408	459	395	444	426
O,N/Total	0.34	0.32	0.39	0.37	0.39	0.41	0.44	0.54	0.57	0.47	0.60	0.44	0.34	0.49	0.50	0.61	0.63	0.64	0.69	0.46	0.56
C/Total	0.66	0.68	0.61	0.63	0.37	0.48	0.56	0.46	0.43	0.53	0.40	0.56	0.66	0.51	0.50	0.39	0.37	0.36	0.31	0.54	0.44
S/Total					0.24	0.11															

(E) 4-ring TWNs (Backbone)

	**Ile**	**Val**	**Leu**	**Phe**	**Cys**	**Met**	**Ala**	**Gly**	**Thr**	**Trp**	**Ser**	**Tyr**	**Pro**	**Hsd**	**Hse**	**Gln**	**Asp**	**Asn**	**Glu**	**Lys**	**Arg**
O	117	106	138	131	132	146	158	148	124	141	140	137	110	133	124	114	107	118	139	143	143
N	44	25	36	41	44	45	41	109	34	32	31	42	49	39	44	44	25	35	42	42	39
C	135	126	138	136	134	145	156	220	104	140	140	134	129	133	134	138	122	134	105	138	132
Total	296	257	312	308	310	336	355	477	262	313	311	313	288	305	302	296	254	287	286	323	314
O,N/Total	0.54	0.51	0.56	0.56	0.57	0.57	0.56	0.54	0.60	0.55	0.55	0.57	0.55	0.56	0.56	0.53	0.52	0.53	0.63	0.57	0.58
C/Total	0.46	0.49	0.44	0.44	0.43	0.43	0.44	0.46	0.40	0.45	0.45	0.43	0.45	0.44	0.44	0.47	0.48	0.47	0.37	0.43	0.42

(F) 4-ring TWNs (Side chain)

	**Ile**	**Val**	**Leu**	**Phe**	**Cys**	**Met**	**Ala**	**Gly**	**Thr**	**Trp**	**Ser**	**Tyr**	**Pro**	**Hsd**	**Hse**	**Gln**	**Asp**	**Asn**	**Glu**	**Lys**	**Arg**
O									75		88	16				41	125	83	93		
N										37				54	57	68		58		21	55
C	177	147	132	155	36	75	101		75	93	34	116	173	100	91	31	29	31	16	100	57
S					111	51															
Total	177	147	132	155	147	126	101		150	130	122	132	173	154	148	140	154	172	109	121	112
O,N/Total									0.50	0.28	0.72	0.12		0.35	0.39	0.78	0.81	0.82	0.85	0.17	0.49
C/Total	1.00	1.00	1.00	1.00	0.24	0.60	1.00		0.50	0.72	0.28	0.88	1.00	0.65	0.61	0.22	0.19	0.18	0.15	0.83	0.51
S/Total					0.76	0.40															

(G) 5-ring TWNs (Backbone + Side chain)

	**Ile**	**Val**	**Leu**	**Phe**	**Cys**	**Met**	**Ala**	**Gly**	**Thr**	**Trp**	**Ser**	**Tyr**	**Pro**	**Hsd**	**Hse**	**Gln**	**Asp**	**Asn**	**Glu**	**Lys**	**Arg**
O	52	56	40	49	53	61	49	58	73	54	96	45	45	53	62	74	90	76	73	52	59
N	13	9	22	16	18	24	11	35	18	28	10	15	9	37	43	18	9	40	10	28	35
C	95	100	94	98	65	74	108	73	58	73	41	76	103	72	67	55	34	51	53	74	57
S					48	24															
Total	160	165	156	163	184	183	168	166	149	155	147	136	157	162	172	147	133	167	136	154	151
O,N/Total	0.41	0.39	0.40	0.40	0.39	0.46	0.36	0.56	0.61	0.53	0.72	0.44	0.34	0.56	0.61	0.63	0.74	0.69	0.61	0.52	0.62
C/Total	0.59	0.61	0.60	0.60	0.35	0.41	0.64	0.44	0.39	0.47	0.28	0.56	0.66	0.44	0.39	0.37	0.26	0.31	0.39	0.48	0.38
S/Total					0.26	0.13															

(H) 5-ring TWNs (Backbone)

	**Ile**	**Val**	**Leu**	**Phe**	**Cys**	**Met**	**Ala**	**Gly**	**Thr**	**Trp**	**Ser**	**Tyr**	**Pro**	**Hsd**	**Hse**	**Gln**	**Asp**	**Asn**	**Glu**	**Lys**	**Arg**
O	52	56	40	49	53	61	49	58	45	54	56	43	45	53	62	56	39	50	32	52	59
N	13	9	22	16	18	24	11	35	18	17	10	15	9	14	17	9	9	14	10	18	19
C	46	46	53	40	55	44	60	73	37	34	30	37	43	38	44	39	26	38	45	45	45
Total	111	111	115	105	126	129	120	166	100	105	96	95	97	105	123	104	74	102	87	115	123
O,N/Total	0.59	0.59	0.54	0.62	0.56	0.66	0.50	0.56	0.63	0.68	0.69	0.61	0.56	0.64	0.64	0.63	0.65	0.63	0.48	0.61	0.63
C/Total	0.41	0.41	0.46	0.38	0.44	0.34	0.50	0.44	0.37	0.32	0.31	0.39	0.44	0.36	0.36	0.38	0.35	0.37	0.52	0.39	0.37

(I) 5-ring TWNs (Side chain)

	**Ile**	**Val**	**Leu**	**Phe**	**Cys**	**Met**	**Ala**	**Gly**	**Thr**	**Trp**	**Ser**	**Tyr**	**Pro**	**Hsd**	**Hse**	**Gln**	**Asp**	**Asn**	**Glu**	**Lys**	**Arg**
O									28		40	2				18	51	26	41		
N										11				23	26	9		26		10	16
C	49	54	41	58	10	30	48		21	39	11	39	60	34	23	16	8	13	8	29	12
S					48	24															
Total	49	54	41	58	58	54	48		49	50	51	41	60	57	49	43	59	65	49	39	28
O,N/Total									0.57	0.22	0.78	0.05		0.40	0.53	0.63	0.86	0.80	0.84	0.26	0.57
C/Total	1.00	1.00	1.00	1.00	0.17	0.56	1.00		0.43	0.78	0.22	0.95	1.00	0.60	0.47	0.37	0.14	0.20	0.16	0.74	0.43
S/Total					0.83	0.44															

(J) 6-ring TWNs (Backbone + Side chain)

	**Ile**	**Val**	**Leu**	**Phe**	**Cys**	**Met**	**Ala**	**Gly**	**Thr**	**Trp**	**Ser**	**Tyr**	**Pro**	**Hsd**	**Hse**	**Gln**	**Asp**	**Asn**	**Glu**	**Lys**	**Arg**
O	11	11	19	12	23	23	19	8	11	11	28	17	8	11	10	19	25	10	28	14	11
N	3	3	4	2	5	5	4	13	6	13	3	4	3	8	12	4	3	17	6	5	6
C	33	25	17	20	12	24	25	16	15	21	13	15	24	10	18	16	12	9	13	16	14
S					20	5															
Total	47	39	40	34	60	57	48	37	32	45	44	36	35	29	40	39	40	36	47	35	31
O,N/Total	0.30	0.36	0.57	0.41	0.47	0.49	0.48	0.57	0.53	0.53	0.70	0.58	0.31	0.66	0.55	0.59	0.70	0.75	0.72	0.54	0.55
C/Total	0.70	0.64	0.43	0.59	0.20	0.42	0.52	0.43	0.47	0.47	0.30	0.42	0.69	0.34	0.45	0.41	0.30	0.25	0.28	0.46	0.45
S/Total					0.33	0.09															

(K) 6-ring TWNs (Backbone)

	**Ile**	**Val**	**Leu**	**Phe**	**Cys**	**Met**	**Ala**	**Gly**	**Thr**	**Trp**	**Ser**	**Tyr**	**Pro**	**Hsd**	**Hse**	**Gln**	**Asp**	**Asn**	**Glu**	**Lys**	**Arg**
O	11	11	19	12	23	23	19	8	9	11	19	16	8	11	10	17	6	8	13	14	11
N	3	3	4	2	5	5	4	13	6	7	3	4	3	1	5	3	3	8	6	4	5
C	13	12	11	5	8	14	10	16	11	8	10	8	8	5	9	11	11	6	11	9	11
Total	27	26	34	19	36	42	33	37	26	26	32	28	19	17	24	31	20	22	30	27	27
O,N/Total	0.52	0.54	0.68	0.74	0.78	0.67	0.70	0.57	0.58	0.69	0.69	0.71	0.58	0.71	0.63	0.65	0.45	0.73	0.63	0.67	0.59
C/Total	0.48	0.46	0.32	0.26	0.22	0.33	0.30	0.43	0.42	0.31	0.31	0.29	0.42	0.29	0.38	0.35	0.55	0.27	0.37	0.33	0.41

(L) 6-ring TWNs (Side chain)

	**Ile**	**Val**	**Leu**	**Phe**	**Cys**	**Met**	**Ala**	**Gly**	**Thr**	**Trp**	**Ser**	**Tyr**	**Pro**	**Hsd**	**Hse**	**Gln**	**Asp**	**Asn**	**Glu**	**Lys**	**Arg**
O									2		9	1				2	19	2	15		
N										6				7	7	1		9		1	1
C	20	13	6	15	4	10	15		4	13	3	7	16	5	9	5	1	3	2	7	3
S					20	5															
Total	20	13	6	15	24	15	15		6	19	12	8	16	12	16	8	20	14	17	8	4
O,N/Total									0.33	0.32	0.75	0.13		0.58	0.44	0.38	0.95	0.79	0.88	0.13	0.25
C/Total	1.00	1.00	1.00	1.00	0.17	0.67	1.00		0.67	0.68	0.25	0.88	1.00	0.42	0.56	0.63	0.05	0.21	0.12	0.88	0.75
S/Total					0.83	0.33															

Table S3. The number of TWNs observed around various atoms of all amino acids in the PDBs. Amino acids are ordered from the most hydrophobic one (Ile, on the left hand side) to the most hydrophilic one (Arg, on the right hand side), according to the Kyte-Doolitle scale.

(A) 4-ring TWNs (Backbone + Side chain)

	**Ile**	**Val**	**Leu**	**Phe**	**Cys**	**Met**	**Ala**	**Gly**	**Thr**	**Trp**	**Ser**	**Tyr**	**Pro**	**His**	**Gln**	**Asp**	**Asn**	**Glu**	**Lys**	**Arg**
O	863	1118	1575	742	187	278	1819	1997	3057	300	3155	2068	1474	372	2121	7128	2343	6629	1180	1006
N	61	130	209	72	24	53	272	315	106	114	174	57	19	827	768	152	822	193	1739	1889
C	114	156	204	103	3	83	141	81	165	47	63	87	264	166	86	62	54	123	258	179
S					45	30														
Total	1038	1404	1988	917	259	444	2232	2393	3328	461	3392	2212	1757	1365	2975	7342	3219	6945	3177	3074
O,N/ Total	0.89	0.89	0.90	0.89	0.81	0.75	0.94	0.97	0.95	0.90	0.98	0.96	0.85	0.88	0.97	0.99	0.98	0.98	0.92	0.94
C/Total	0.11	0.11	0.10	0.11	0.01	0.19	0.06	0.03	0.05	0.10	0.02	0.04	0.15	0.12	0.03	0.01	0.02	0.02	0.08	0.06
S/Total					0.17	0.07														

(B) 4-ring TWNs (Backbone)

	**Ile**	**Val**	**Leu**	**Phe**	**Cys**	**Met**	**Ala**	**Gly**	**Thr**	**Trp**	**Ser**	**Tyr**	**Pro**	**His**	**Gln**	**Asp**	**Asn**	**Glu**	**Lys**	**Arg**
O	863	1118	1575	742	187	278	1819	1997	1092	300	1161	666	1474	372	692	1212	950	1149	1180	1006
N	61	130	209	72	24	53	272	315	106	36	174	57	19	72	98	152	153	193	156	98
C	0	5	5	5	1	2	16	81	9	2	19	5	21	3	6	10	13	10	7	12
Total	924	1253	1789	819	212	333	2107	2393	1207	338	1354	728	1514	447	796	1374	1116	1352	1343	1116
O,N/ Total	1.00	1.00	1.00	0.99	1.00	0.99	0.99	0.97	0.99	0.99	0.99	0.99	0.99	0.99	0.99	0.99	0.99	0.99	0.99	0.99
C/Total	0.00	0.00	0.00	0.01	0.00	0.01	0.01	0.03	0.01	0.01	0.01	0.01	0.01	0.01	0.01	0.01	0.01	0.01	0.01	0.01

(C) 4-ring TWNs (Side chain)

	**Ile**	**Val**	**Leu**	**Phe**	**Cys**	**Met**	**Ala**	**Gly**	**Thr**	**Trp**	**Ser**	**Tyr**	**Pro**	**His**	**Gln**	**Asp**	**Asn**	**Glu**	**Lys**	**Arg**
O									1965		1994	1402			1429	5916	1393	5480		
N										78				755	670		669		1583	1791
C	114	151	199	98	2	81	125		156	45	44	82	243	163	80	52	41	113	251	167
S					45	30														
Total	114	151	199	98	47	111	125		2121	123	2038	1484	243	918	2179	5968	2103	5593	1834	1958
O,N/ Total									0.93	0.63	0.98	0.94		0.82	0.96	0.99	0.98	0.98	0.86	0.91
C/Total	1.00	1.00	1.00	1.00	0.04	0.73	1.00		0.07	0.37	0.02	0.06	1.00	0.18	0.04	0.01	0.02	0.02	0.14	0.09

	Ile	Val	Leu	Phe	Cys	Met	Ala	Gly	Thr	Trp	Ser	Tyr	Pro	His	Gln	Asp	Asn	Glu	Lys	Arg
S/Total					0.96	0.27														

(D) 5-ring TWNs (Backbone + Side chain)

	Ile	Val	Leu	Phe	Cys	Met	Ala	Gly	Thr	Trp	Ser	Tyr	Pro	His	Gln	Asp	Asn	Glu	Lys	Arg
O	307	447	491	227	53	123	743	669	1185	118	1146	756	564	169	765	2775	788	2600	478	397
N	31	34	79	23	2	10	89	81	36	31	47	15	5	261	264	46	307	51	568	618
C	34	46	60	32	3	30	61	22	52	18	17	18	74	49	24	15	12	44	66	51
S					13	15														
Total	372	527	630	282	71	178	893	772	1273	167	1210	789	643	479	1053	2836	1107	2695	1112	1066
O,N/ Total	0.91	0.91	0.90	0.89	0.77	0.75	0.93	0.97	0.96	0.89	0.99	0.98	0.88	0.90	0.98	0.99	0.99	0.98	0.94	0.95
C/Total	0.09	0.09	0.10	0.11	0.04	0.17	0.07	0.03	0.04	0.11	0.01	0.02	0.12	0.10	0.02	0.01	0.01	0.02	0.06	0.05
S/Total					0.18	0.08														

(E) 5-ring TWNs (Backbone)

	Ile	Val	Leu	Phe	Cys	Met	Ala	Gly	Thr	Trp	Ser	Tyr	Pro	His	Gln	Asp	Asn	Glu	Lys	Arg
O	307	447	491	227	53	123	743	669	442	118	401	234	564	169	248	471	319	454	478	397
N	31	34	79	23	2	10	89	81	36	3	47	15	5	23	33	46	66	51	41	32
C	1	0	1	2	0	3	11	22	3	2	8	0	7	6	2	3	3	3	1	2
Total	339	481	571	252	55	136	843	772	481	123	456	249	576	198	283	520	388	508	520	431
O,N/ Total	1.00	1.00	1.00	0.99	1.00	0.98	0.99	0.97	0.99	0.98	0.98	1.00	0.99	0.97	0.99	0.99	0.99	0.99	1.00	1.00
C/Total	0.00	0.00	0.00	0.01	0.00	0.02	0.01	0.03	0.01	0.02	0.02	0.00	0.01	0.03	0.01	0.01	0.01	0.01	0.00	0.00

(F) 5-ring TWNs (Side chain)

	Ile	Val	Leu	Phe	Cys	Met	Ala	Gly	Thr	Trp	Ser	Tyr	Pro	His	Gln	Asp	Asn	Glu	Lys	Arg
O									743		745	522			517	2304	469	2146		
N										28				238	231		241		527	586
C	33	46	59	30	3	27	50		49	16	9	18	67	43	22	12	9	41	65	49
S					13	15														
Total	33	46	59	30	16	42	50		792	44	754	540	67	281	770	2316	719	2187	592	635
O,N/ Total									0.94	0.64	0.99	0.97		0.85	0.97	0.99	0.99	0.98	0.89	0.92
C/Total	1.00	1.00	1.00	1.00	0.19	0.64	1.00		0.06	0.36	0.01	0.03	1.00	0.15	0.03	0.01	0.01	0.02	0.11	0.08
S/Total					0.81	0.36														

(G) 6-ring TWNs (Backbone + Side chain)

	**Ile**	**Val**	**Leu**	**Phe**	**Cys**	**Met**	**Ala**	**Gly**	**Thr**	**Trp**	**Ser**	**Tyr**	**Pro**	**His**	**Gln**	**Asp**	**Asn**	**Glu**	**Lys**	**Arg**
O	105	176	173	157	67	36	324	312	813	47	505	316	148	48	354	1738	430	1168	115	127
N	9	17	17	2	0	3	44	43	12	31	19	11	4	109	142	14	248	12	371	306
C	11	6	16	36	0	25	18	10	26	2	15	5	21	16	34	14	17	11	26	17
S					3	4														
Total	125	199	206	195	70	68	386	365	851	80	539	332	173	173	530	1766	695	1191	512	450
O,N/ Total	0.91	0.97	0.92	0.82	0.96	0.57	0.95	0.97	0.97	0.98	0.97	0.98	0.88	0.91	0.94	0.99	0.98	0.99	0.95	0.96
C/Total	0.09	0.03	0.08	0.18	0.00	0.37	0.05	0.03	0.03	0.03	0.03	0.02	0.12	0.09	0.06	0.01	0.02	0.01	0.05	0.04
S/Total					0.04	0.06														

(H) 6-ring TWNs (Backbone)

	**Ile**	**Val**	**Leu**	**Phe**	**Cys**	**Met**	**Ala**	**Gly**	**Thr**	**Trp**	**Ser**	**Tyr**	**Pro**	**His**	**Gln**	**Asp**	**Asn**	**Glu**	**Lys**	**Arg**
O	105	176	173	157	67	36	324	312	220	47	148	92	148	48	96	146	139	140	115	127
N	9	17	17	2	0	3	44	43	12	17	19	11	4	11	17	14	107	12	7	7
C	0	0	0	0	0	2	1	10	1	1	1	0	1	0	1	0	2	1	0	0
Total	114	193	190	159	67	41	369	365	233	65	168	103	153	59	114	160	248	153	122	134
O,N/ Total	1.00	1.00	1.00	1.00	1.00	0.95	1.00	0.97	1.00	0.98	0.99	1.00	0.99	1.00	0.99	1.00	0.99	0.99	1.00	1.00
C/Total	0.00	0.00	0.00	0.00	0.00	0.05	0.00	0.03	0.00	0.02	0.01	0.00	0.01	0.00	0.01	0.00	0.01	0.01	0.00	0.00

(I) 6-ring TWNs (Side chain)

	**Ile**	**Val**	**Leu**	**Phe**	**Cys**	**Met**	**Ala**	**Gly**	**Thr**	**Trp**	**Ser**	**Tyr**	**Pro**	**His**	**Gln**	**Asp**	**Asn**	**Glu**	**Lys**	**Arg**
O									593		357	224			258	1592	291	1028		
N										14				98	125		141		364	299
C	11	6	16	36	0	23	17		25	1	14	5	20	16	33	14	15	10	26	17
S					3	4														
Total	11	6	16	36	3	27	17		618	15	371	229	20	114	416	1606	447	1038	390	316
O,N/ Total									0.96	0.93	0.96	0.98		0.86	0.92	0.99	0.97	0.99	0.93	0.95
C/Total	1.00	1.00	1.00	1.00	0.00	0.85	1.00		0.04	0.07	0.04	0.02	1.00	0.14	0.08	0.01	0.03	0.01	0.07	0.05
S/Total					1.00	0.15														

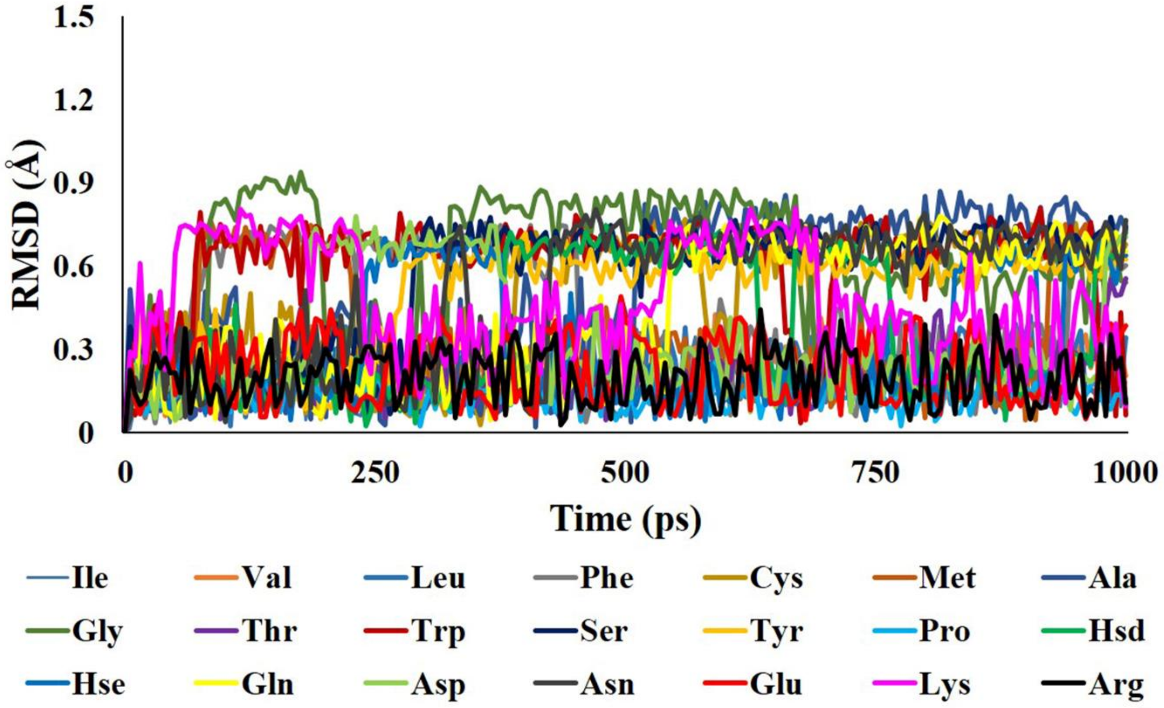

Figure S1. RMSD plot for the backbone atoms of amino acids from the initial structures throughout the 1 ns MD simulation as a function of time.

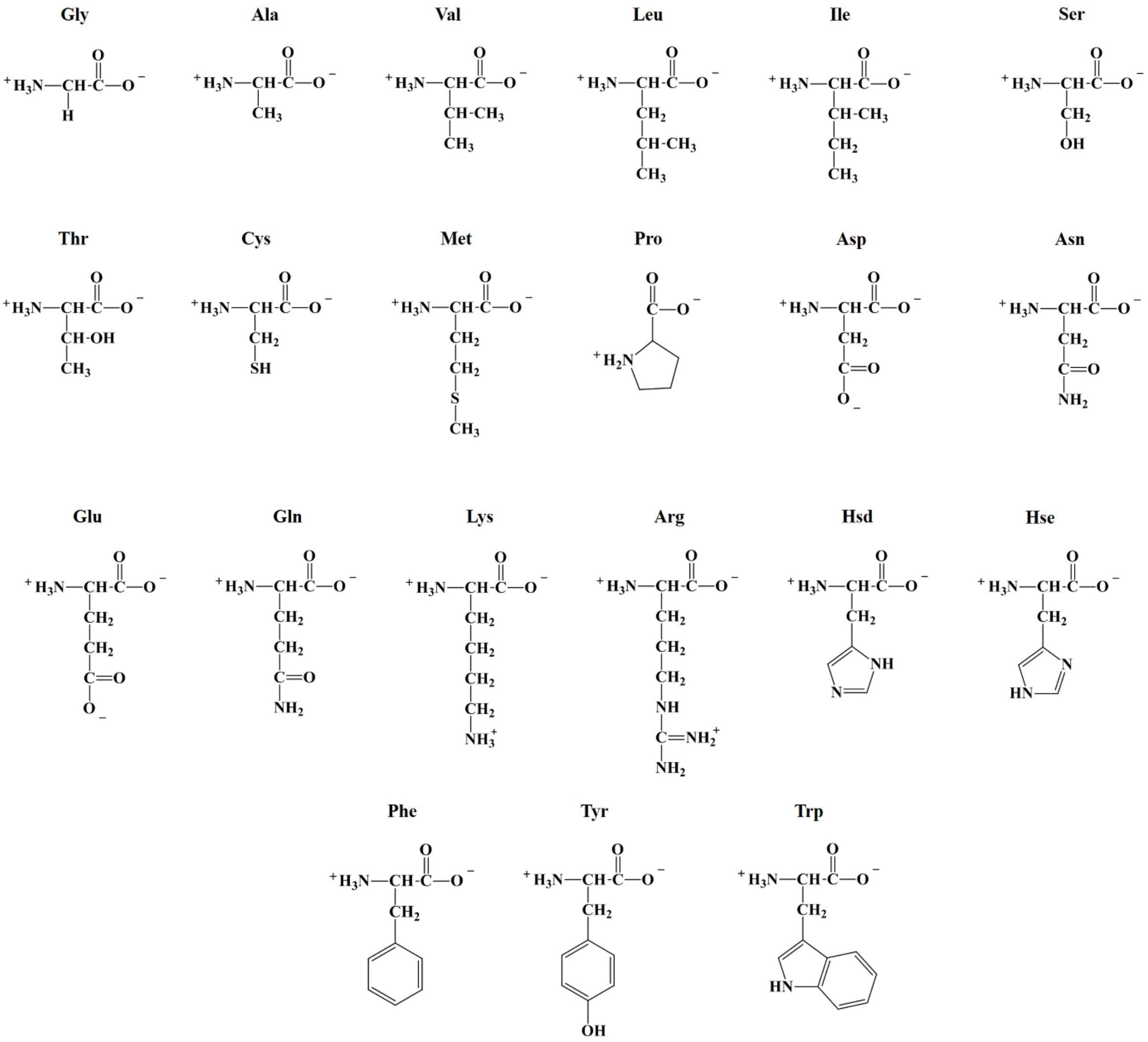

Figure S2. Structures of the amino acids studied in the present work.
